# Impact of self-assessment on dental student’s performance in pre-clinical conservative dentistry course

**DOI:** 10.1186/s12903-024-04140-w

**Published:** 2024-05-22

**Authors:** Priya Mittal, Ganesh R. Jadhav, Mansing Pawar, Sitikantha Banerjee, Sneha Wangaskar, Marco Di Blasio, Gabriele Cervino, Giuseppe Minervini

**Affiliations:** 1 Department of Conservative Dentistry and Endodontics, Swargiya Dadasaheb Kalmegh Smruti Dental College & Hospital, Nagpur, India; 2 Department of Dentistry, AIIMS Nagpur, Nagpur, India; 3 International Collaborations, Krishna Vishwa Vidyapeeth, Karad, Maharashtra India; 4 Department of Community Medicine, AIIMS Jammu, Jammu, India; 5 ICSSR Project, Dept of Community Medicine, AIIMS, Nagpur, India; 6 Department of Biomedical Surgical and Dental Sciences, University of Milan, Milan, Italy; 7 School of Dentistry, Department of Biomedical and Dental Sciences and Morphofunctional Imaging, University of Messina, via Consolare Valeria, 1, Messina, 98125 Italy; 8 Saveetha Dental College and Hospitals, Saveetha Institute of Medical and Technical Sciences (SIMATS), Saveetha University, Chennai, India; 9 Multidisciplinary Department of Medical-Surgical and Odontostomatological Specialties, University of Campania “Luigi Vanvitelli”, Naples, 80121 Italy

**Keywords:** Conservative, Dentistry student, Self-assessment

## Abstract

**Background:**

Self-assessment (SA) is an interactive course that endorses the accomplishment of learning objectives through learners’ identification of insufficiencies in their didactic knowledge and pre-clinical skills. This study was planned to determine whether there is any improvement in the faculty assessment (FA) score following the implementation of SA in the Pre-clinical Conservative Dentistry Course.

**Methods:**

Fifty-four first-semester dental students were given an introductory lecture followed by a demonstration for Class I Cavity Preparation in typhodont mandibular first molar. At the end of the demonstration, the Scoring Rubric (SR) was explained point-wise in the prepared cavities. During the next session, all students performed Class I cavity preparation and they were given an assessment sheet to enter their scores (SA1). All teeth were evaluated by the Grading Faculties in a blinded manner (FA1). Each participant was explained the difference in their respective SA1 from FA1 and their queries were resolved individually. During the next sessions, Students and Grading Faculties followed the same protocol and scores were recorded as SA2, FA2, SA3 and FA3.

**Results:**

The mean score of SA1 was significantly higher than that of FA1 (*p* < 0.001). However, no significant difference was obtained between SA and FA in the second (*p* = 0.352) and third (*p* = 0.434) assessments. In contrast with first assessment, mean marks obtained in FA were higher compared to SA in both second and third assessments. There was a statistically significant improvement in mean marks obtained by the students over time (*p* < 0.001).

**Conclusion:**

SA endorsed student-faculty communication and enhanced student’s poise and technical skills in operative pre-clinical dentistry.

## Introduction

Dental education (DE) is an amalgamation of theoretical information as well as the progress of fine manual skills [1]. Theoretical information, comprising of obtaining information on the physiology and pathology of oral structure, is one of the essentials for unhindered pre-clinical as well as clinical works. This information can be used to examine, diagnose, and plan a treatment through self-direction and faculty feedback. Dental students have little difficulty with manual skill component of DE as the Pre-clinical (PC) component involving manual dexterity is a new skill set for second-year undergraduates [2–9]. Unacquaintedness with the PC work leads students to seek guidance from their instructors without first reflecting on their work. Self-assessment (SA) is an interactive course that endorses the accomplishment of learning objectives through learners’ identification of insufficiencies in their didactic knowledge and PC skills [1]. Dental student’s self-assessment (SA) abilities are essential for constant learning to progress their skills based on their knowledge [10–15]. SA reflects student’s ability to take responsibility for their learning and acknowledges their skills and learning needs. Feedback from faculty is generally regarded as an important aspect to improve student’s knowledge and skills [16]. Faculty feedback following SA helps students familiarize themselves with faculty expectations and comprehend course aims and assessment norms [17–19]. The literature revealed that constructive faculty feedback favourably impacts student’s self-confidence; and hence, SA has been accepted in several fields [20–26]. Wettergreen et al. conducted a study to assess FA and SA during the clinical case discussions in a pharmacotherapy capstone course. According to their results, SA reinforced with FA improved the students’ performance [23].

At Kalmegh College, three faculty assessments (FA) are required for appearing in the first internal assessment PC conservative dentistry (PCCD) Examination which consists of Class I Cavity Preparation for Amalgam. In the second-year PC Conservative Dentistry Course, SA was introduced as a new prerequisite in 2022 as a precursor to the FA to help students to recognize their strengths and deficits in PC Class I Cavity Preparation. During SA, students reflect on their knowledge and performance to assign themselves grades for the procedures. Later, they are also evaluated by the faculty and given feedback on their performance. A passing grade on a SA is determined by the FA.

The design of current study integrated a well-established “glance and grade” method along with a rubric and SA methods to evaluate the practical performance of dental students. Here an important concept in DE was addressed that improves the knowledge and performance of dental students in a PC setting. The structured rubrics not only guides students in recognising and addressing their areas of weakness but also helps teachers in effective teaching. Students’ capacity to accurately identify their own shortcomings can help them set and achieve effective learning objectives. Hence this study was planned to determine whether there is any improvement in the FA score following the implementation of SA in the PCCD Course. Present authors hypothesised (H_0_) that there will be no improvement in the FA score following the implementation of SA in the PCCD Course.

## Methods

Approval for this prospective cohort study was given by the Institutional Review Board (SDKSDCH/IEC/FACULTY/089/2022). The study was carried out in accordance with the Helsinki Declaration of 1975, as revised in 2000. Informed consent was obtained from all the participants and/or legal guardians for the study. PCCD Course is a second-year, two-semester course. The first and second semesters include Class I and Class II Cavity Preparations for Amalgam along with base application and restoration respectively. In this study, first-semester students (*n* = 54; male 15; female 39) were evaluated for Class I cavity preparation using a newly designed scoring rubric (SR) (Table 1). This scoring rubric (SR) is a collaborative effort of four senior faculties from the Department of Conservative Dentistry and Endodontics from different institutions and is based on their previous experience in assessing student’s performance in the PC Examination. Grading faculty members were two full-time faculty members (PM, GJ) who were calibrated with a calibration exercise. Agreement between both examiners when assessing cavity preparation was made. SA and FA scores were the variables of the study.

**Table 1:** Class I Cavity Preparation Scoring Rubric

CLASS - I CAVITY PREPARATION RUBRIC			
**OUTLINE FORM**			
**Cavity Outline**	2	1	0
	Inclusion of all fissures Not extending > 1/2 of cuspal incline	Inclusion/Exclusion of all fissures Extending > 1/2 of cuspal incline but < 2/3 of cuspal incline	Inclusion/ Exclusion of all fissures Extending > 2/3 of cuspal incline
**RESISTANCE FORM**			
**Floor of cavity**		1	0
		Flat Pulpal floor	Pulpal floor is not flat
**Internal line angle** **(8 line angles)**		1	0
		Rounded internal line angles	Sharp internal line angles
**Depth of cavity**	2	1	0
	1.5-2 mm	Deviation of 0.5 mm from ideal depth	Deviation of > 0.5 mm from ideal depth
**Marginal ridge**	2	1	0
	2 mm in molars, 1.6 mm in premolars	± 0.5 mm deviation from ideal dimensions	> 0.5 mm deviation from ideal dimensions
**RETENTION FORM**			
**Walls direction (facial & lingual)**	2	1	0
	Convergent	Parallel	Divergent
**CONVENIENCE FORM**			
**Width of cavity**		1	0
		1/4th -1/5th intercuspal distance	Deviation from normal dimensions

**Fig. 1:**
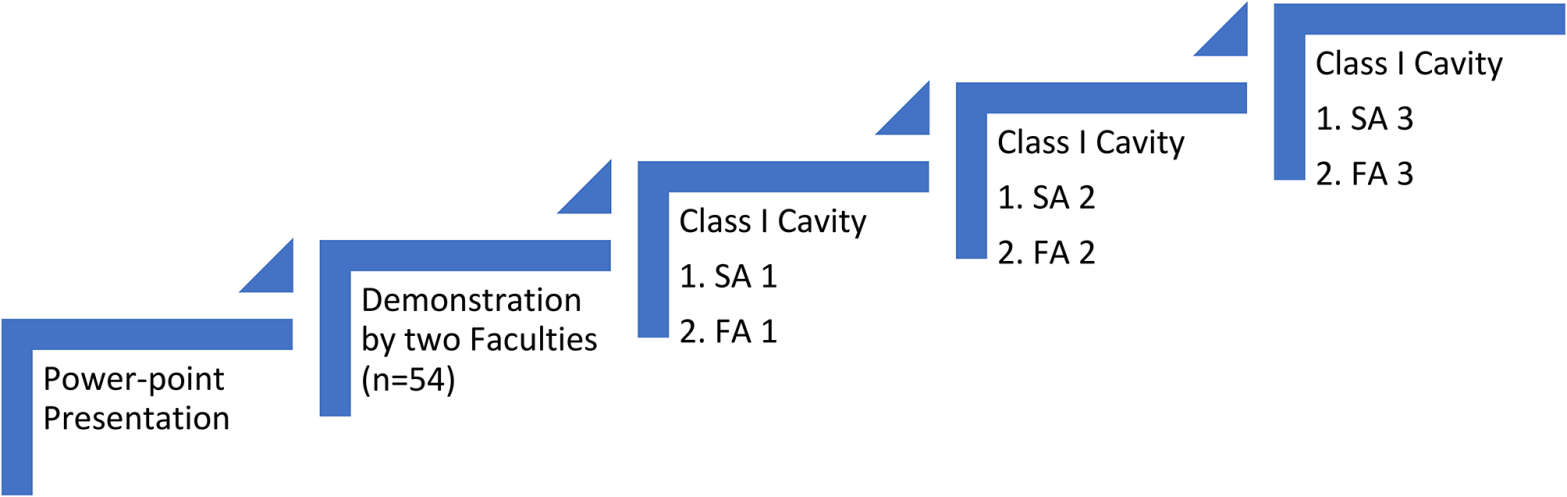
Different steps in the study

Different steps in the study are depicted in Fig. 1. All students were given an introductory lecture about Class I cavity preparation via PowerPoint Presentation. Later, students received a demonstration for conventional Class I cavity preparation on typhodont mandibular first molar tooth from the faculty. At the end of the demonstration, the SR was explained point-wise in the prepared cavities. During the first session, all students (*n* = 54) executed Class I cavity preparation in typhodont mandibular first molar tooth (Frasaco-USA) of articulated jaw model (TRU LON study model, Jayna industries, Ghaziabad, India) using the cavity preparation armamentarium. Micromotor (NSK, Nakanishi Inc)) and ISO # 245 tungsten carbide bur (S.S. White, New York, USA) were used by students. After cavity preparation, students were given an assessment sheet to enter their scores (SA1). All teeth were collected, labelled and evaluated by both the grading faculties in a blinded manner using William’s Periodontal Probe (Hu-Friedy Mfg. Co., LLC, UK) and the average of their scores (from both the faculties) was considered the final score [Faculty Assessment Score 1 (FA1)]. These scores (SA1, FA1) were treated as a baseline performance because they represented the student’s first experience in a PC practical class. Each participant was explained the difference in their respective SA1 from FA1 and their queries were resolved individually. Students prepared cavities in the next two sessions. Students and grading faculties followed the same protocol and scores were recorded as SA2, FA2, SA3, FA3. All the scores were entered in an Excel Sheet (Microsoft 365, Redmond, Washington, United States) and compared with each other. Cavity Preparation was graded for a maximum score of 11 as exceptional (scores 9–11), minor errors but meets expectations (scores 6–8), minimally acceptable (scores 3–5), and unsatisfactory with major errors that need repetition (score 0–2). During the SA exercises, students perform the procedure and demonstrate knowledge and skill independently, with faculty feedback and assistance as needed.

### Statistical analysis

The data were entered in Microsoft excel, and *SPSS* software (SPSS 23.0; IBM, Armonk, NY, USA) was used for statistical analysis. The mean marks obtained by the students were compared between SA and FA individually for each of the three assessments. The correlation of mean marks was calculated for different time points. The change of mean FA scores over three time points was calculated using repeated measure ANOVA, and the pairwise correction was run using Greenhouse Geisser statistics. SA and FA scores were compared separately at three different time points using a paired sample t-test.

## Results

The mean score of SA1 was significantly higher than that of FA1 (*p* < 0.001)). However, no significant difference was obtained between SA and FA in the second (*p* = 0.352) and third (*p* = 0.434). In contrast with first assessment, mean marks obtained in FA (8.28) were higher compared to SA in both second and third assessments (Table 2). The correlation of marks obtained through student and faculty assessment at three different time points was analysed. Pearson correlation coefficient (r) was found to be on the higher side (0.7) in third assessment, compared to first and second assessments. However, a statistically significant correlation was also found in second assessment (*p* = 0.026) (Table 3). The findings suggest that there was a statistically significant improvement in mean marks obtained by the students over time (*p* < 0.001). The mean marks were significantly higher at each time point, compared to the preceding time points (*p* < 0.001). (Table 4)

**Table 2:** Mean Difference of the marks obtained by the students using self and faculty assessment (*n* = 54)

Assessment	Marks Obtained by the students Mean (SD)	Mean Difference (95% CI)	*p* value*
Student Assessment-1	6.72 (1.43)	5.09 (4.26, 5.56)	**0.0001**
Faculty Assessment-1	1.63 (0.89)		
Student Assessment-2	4.13 (1.30)	-0.29 (-0.93, 0.34)	0.352
Faculty Assessment-2	4.43 (1.57)		
Student Assessment-3	8.15 (1.61)	-0.13 (-0.45, 0.19)	0.434
Faculty Assessment-3	8.28 (1.47)		

*Paired sample t-test was used

**Table 3:** Correlation of mean marks obtained by students (*n* = 54)

Assessments	Correlation Coefficient*	*p* value
Student Assessment-1 and Faculty Assessment-1	-0.038	0.786
Student Assessment-2 and Faculty Assessment-2	-0.30	**0.026**
Student Assessment-3 and Faculty Assessment-3	0.70	**0.0001**

*Pearson correlation coefficient used

**Table 4:** Change of mean faculty assessment marks over three-time point (*n* = 54)

Assessment	Marks Obtained by the students Mean (SD)	F value	*p* value*	Effect Size (Partial Eta Square)
Faculty Assessment-1	1.63 (O.89)	478.5	**< 0.0001**	0.9
Faculty Assessment-2	4.43 (1.57)			
Faculty Assessment-3	8.28 (1.47)			
Pairwise comparison				
Assessment pairs	Mean Difference	95% CI		*p* value
Faculty Assessment-1 and Faculty Assessment-2	-2.79	-3.30, -2.29		**< 0.0001**
Faculty Assessment-2 and Faculty Assessment-3	-3.86	-4.47, -3.23		**< 0.0001**
Faculty Assessment-1 and Faculty Assessment-3	-6.65	-7.11, -6.19		**< 0.0001**

*Repeated measure ANOVA with pairwise correction was used using Greenhouse Geisser statistics

## Discussion

The aim of this study was to determine whether there is any improvement in the assessment score of the PCCD student for Class I cavity preparation following the implementation of SA and scoring rubric. The results of study show improvement in scores after each assessment. Thus, the assumed null hypothesis (H_0_) that there will be no improvement in the FA score following the implementation of SA in the PCCD Course was rejected. The competence of dental students to self-evaluate their work is crucial for constant learning as well as upgrading their knowledge and skills [27]. This study demonstrates the role of SA and faculty feedback on improving dental student’s clinical skills in a PCCD course. Scores obtained were meaningfully upgraded after student’s self-assessments and skills were strengthened through this process. SA presents a learning prospect for participants to shape confidence, gain experience, and advance insight into the DE process. SA gives students a chance to better comprehend the principles and objectives of the different procedures and fully test their skills without the risk of negative implications. Moreover, SA makes students understand the difference between error and ideal performance. Our study found that students had a higher score on final faculty assessment (FA 3) due to the effective implementation of the SA exercise. This finding is consistent with the results of other studies [28–34].

Our study showed a significant difference between SA1 and FA1. This finding was consistent with previous studies that stated the limited predictive value of students because they are inclined to overestimate their own skills [24, 26, 35–39]. However, there was no significant difference between SA2-FA2 and SA3-FA3 scores, likely due to the open and crystal-clear feedback mechanism between the faculties and students during the SA exercise. Additionally, the significant improvement in Final Grades (in both SA3 and FA3) was likely due to such endeavours’ better-empowering students to differentiate mistakes from ideal performance. The differences between dental student’s SA of their ability and FA decreased from the first to the third sessions with more exercises and practical training, developing better self-insight [40]. In the authors’ experience, participants who successfully completed SA1, approached subsequent sessions more positively and with less concern for poor performance. This finding was consistent with the previous studies that reported a positive correlation between the improvement in SA and higher scores in the examination [31].

Student and Faculty response to the SA program has been overwhelmingly positive. Firstly, it was perceived as an extra task by students and faculties. Once initiated, the SA program swiftly became a valued PCCD exercise and a teaching tool. Moreover, it improved the discussion between students and faculty as students do not have to fear implications of failure or a poor grade. Filling out the evaluation forms and following the grading criteria provide an objective assessment by faculty and an educational goal based on SA by the student.

Faculty Feedback given during SA prepares the student with an outline of what is expected during the cavity preparation and, by identifying areas of deficient preparedness or knowledge, creates a focused application of readiness in areas needing upgrading. Furthermore, the process allows each student to undergo self-evaluation as many as three times to obtain adequate feedback. Some students need self-assessment once, whereas others require more attempts to develop the knowledge and skills they need to successfully complete the minimally acceptable score (scores 3–5). This study found a positive correlation between improved recognition skills and a corresponding performance improvement, similar to that published in previous studies [41, 42]. Dental students obviously progress through repeated experiences and prolonged time irrespective of the formal assessment methods. However, providing self-reflection instructions helps participants identify areas of shortcomings and gives them insight into expectations on formal assessments. Now in modern dental practice, virtual reality assisted learning (VRAL) is emerging new technology which uses an artificial reality or environments, with which the user can interact. During VRAL, operators use haptic devices for dental procedures in a virtual environment, with instructions and feedback received from the computer. By applying the same principles to restorative dentistry, Virtual reality (VR) and augmented reality (AR) simulations can help students improve manual dexterity during Class I and Class II cavity preparations [43].

Rubrics are known to the educational community as a means of communicating educational goals to students, providing targeted feed-back and assessing the results. Rubrics provide students with the criteria dimensions that demonstrate the expectations of a task assigned to them and the description of the performance levels for each dimension [44]. By using rubrics in self- assessment, it is possible to measure the level of learning and performance of students in different learning areas [45, 46]. In the present study, the students of the experimental groups used rubrics which was a validated tool for self-assessment as well as for faculties feedback.

### Limitations

To evaluate practical skills of students, various digital simulation systems are available like DentSim (DenX, Jerusalem, Israel), PREP assistant (KaVo, Biberach, Germany) and prep Check (Dentsply Sirona, Wals, Austria). These systems provide feedback with increased reliability during the learning process. So, further studies can be planned to incorporate digital system along with faculty feedback for betterment of undergraduate students. Moreover, findings of the present study are limited to pre-clinical work and future studies can be planned for clinical work.

## Conclusion

This study showed that there is an improvement in faculty assessment scores following the incorporation of self-assessment in the Pre-clinical Conservative Dentistry Course. Moreover, the integration of scoring rubric with self-assessment for evaluation of Class I cavity preparation improved the skillset as well as understanding of the participated dental students.

## Data Availability

The data will be available on reasonable request from the corresponding author.
